# Investigation of simulated microgravity effects on *Streptococcus mutans* physiology and global gene expression

**DOI:** 10.1038/s41526-016-0006-4

**Published:** 2017-01-12

**Authors:** Silvia S. Orsini, April M. Lewis, Kelly C. Rice

**Affiliations:** 1 Department of Microbiology and Cell Science, Institute of Food and Agricultural Sciences, University of Florida, Gainesville, FL 32611-0700 USA; 2 Brammer Bio, Alachua, Florida, 32615 USA

## Abstract

Astronauts have been previously shown to exhibit decreased salivary lysozyme and increased dental calculus and gingival inflammation in response to space flight, host factors that could contribute to oral diseases such as caries and periodontitis. However, the specific physiological response of caries-causing bacteria such as *Streptococcus mutans* to space flight and/or ground-based simulated microgravity has not been extensively investigated. In this study, high aspect ratio vessel *S. mutans* simulated microgravity and normal gravity cultures were assessed for changes in metabolite and transcriptome profiles, H_2_O_2_ resistance, and competence in sucrose-containing biofilm media. Stationary phase *S. mutans* simulated microgravity cultures displayed increased killing by H_2_O_2_ compared to normal gravity control cultures, but competence was not affected. RNA-seq analysis revealed that expression of 153 genes was up-regulated ≥2-fold and 94 genes down-regulated ≥2-fold during simulated microgravity high aspect ratio vessel growth. These included a number of genes located on extrachromosomal elements, as well as genes involved in carbohydrate metabolism, translation, and stress responses. Collectively, these results suggest that growth under microgravity analog conditions promotes changes in *S. mutans* gene expression and physiology that may translate to an altered cariogenic potential of this organism during space flight missions.

## Microbiology: Cavities-causing bacteria altered by space-like conditions

The gene expression patterns, metabolism and physiology of tooth cavities-causing microbes change in a space-like gravity environment. These findings could help explain why astronauts are at a greater risk for dental diseases when in space. Kelly Rice and colleagues from the University of Florida, Gainesville, USA, cultured *Streptococcus mutans* bacteria under simulated microgravity and normal gravity conditions. The bacteria grown in microgravity were more susceptible to killing with hydrogen peroxide, tended to aggregate in more compact cellular structures, showed changes in their metabolite profile and expressed around 250 genes at levels that were either much higher or lower than normal gravity control cultures. These genes included many involved in carbohydrate metabolism, protein production and stress responses. The observed changes collectively suggest that space flight and microgravity could alter the cavities-causing potential of *S. mutans*.

## Introduction

*Streptococcus mutans* is a primary causative agent of dental caries, demonstrated by its isolation from carious tooth lesions in humans,^1^ its ability to initiate caries in germ-free rodent models of infection,^2^ and the established link between high levels of this bacterium in the oral cavity and active caries.^3^ Although preventable by fluoridation of water and various dental procedures,^4^, caries remains the most common chronic disease among children, and remains untreated in up to 30% of adults over 35 years-old.^5^ *S. mutans* transient bacteremias can also initiate endocarditis in at-risk patient groups.^6^ Successful colonization of the oral cavity and persistence in multi-species dental plaque by *S. mutans* is inherently dependent on both its ability to form biofilm and its rapid metabolism of carbohydrates by the glycolytic pathway. The low pH that results from accumulation of acidic metabolic end-products favors the growth of *S. mutans* and other acid-tolerant species relative to healthy plaque microflora, as well as the eventual demineralization of tooth enamel and the development of caries. Adherence of *S. mutans* to the tooth surface and subsequent biofilm development are mediated by several bacterial factors, including production of sucrose-dependent extracellular glucan polymers by glucosyltransferase enzymes,^7^ specific cell surface adhesins,^8, 9^ the major autolysin AtlA,^10^ the quorum-sensing competence (*com*) system,^11^ and extracellular DNA.^12^

To protect the health of astronauts during space flight missions, it is critical to understand the effects of this environment on both host biology and the host microbiota. Simulated microgravity exposure has been shown to cause increased mandibular and alveolar bone loss^13^ and decreased saliva flow,^14^ significant predisposing host factors that could contribute to development of caries and/or periodontal disease. Traditionally, the approach to maintaining astronaut oral health during space flight missions has been routed in preventative dentistry, whereby astronauts are subjected to rigorous preflight dental screening and treatment.^15^ However, in the face of future long-term space flight missions, research and discovery of new methods for maintaining oral hygiene and dental health under a microgravity environment are required.^15^ Previous studies on the effects of spaceflight on the oral microbiota have primarily focused on culture-dependent methods of quantifying the numbers of oral bacteria in human subjects before and after spaceflight.^16–18^ Furthermore, astronauts have been shown to exhibit decreased salivary lysozyme and increased dental calculus and gingival inflammation in response to space flight.^16^ However, assessment of the physiology and virulence potential of oral pathogens such as *S. mutans* under controlled microgravity analog conditions has been relatively lacking. In this respect, rotating wall vessel bioreactors are a common technology used to grow bacteria under microgravity analog conditions in ground-based studies. When completely filled with media (lacking air bubbles or “head space”) and rotated on the axis perpendicular to the Earth’s gravitational vector, these reactors maintain a low-shear environment (<1 dyn/cm^2^) whereby gravitational vectors are randomized over the surface of the cells or particles contained within the vessel.^19^ As such, this simulated environment models “weightlessness” by counteracting gravitational forces that would otherwise promote cellular sedimentation.^19,20^ The low-shear force experienced in this microgravity analog system boasts the additional advantage of mimicking the low-shear force of saliva flow (<0.8 dyn/cm^2^) experienced by plaque bacteria in the oral cavity.^21^ In this study, high-aspect rotating vessels (HARVs) were therefore used to ascertain the effects of simulated microgravity on *S. mutans* gene expression, physiology, oxidative stress resistance, and competence, using both culture-dependent and “-omic” (metabolomics and RNA-seq) approaches.

## Results

### *S. mutans* HARV growth curves and cell aggregation

To probe the effect of simulated microgravity growth on *S. mutans*, continuous growth curves (whereby cell samples were removed from the same HARV at different time points) were performed (*n* = 3 independent experiments each) in biofilm media (BM) containing 11 mM glucose and 10 mM sucrose over a 24 h period. In this analysis, no statistically significant differences in CFU/ml at each time point were observed (Supplemental Fig. S1). An initial decrease in CFU/ml was observed in both the simulated microgravity and normal gravity HARV cultures between *t* = 0 (time of inoculation) and *t* = 2 h growth, which was likely a function of sucrose-dependent cell aggregation. Very small differences in cell viability patterns in early stationary phase (6–8 h growth) suggest that the simulated microgravity HARV culture may have entered stationary phase slightly sooner than the normal gravity HARV culture in this experiment. As well, the simulated microgravity culture persisted in stationary phase until approximately 12 h growth, followed by a somewhat rapid death phase, with a 1-log loss of viability between 12 and 24 h growth. In contrast, the viability of normal gravity culture underwent a more gradual decrease in cell viability between 12 and 24 h of growth.

**Fig. 1:**
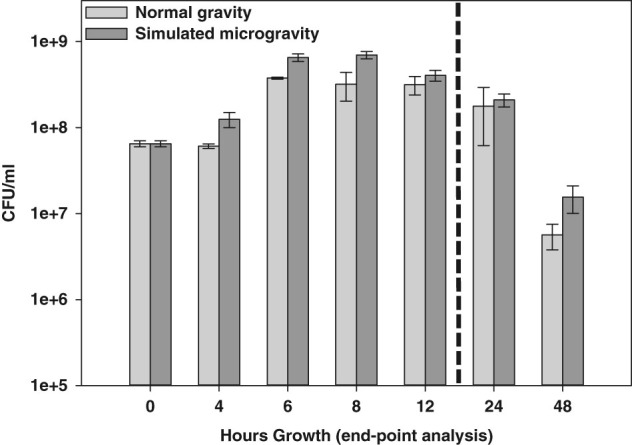
End-point growth curve analysis of *S. mutans* simulated microgravity and normal gravity HARV cultures. Simulated microgravity (*dark gray bars*) and normal gravity (*light gray bars*) HARV end-point growth was measured by serial dilution and CFU/ml plating at *t* = 0 (time of inoculation; average of *n* = 4 independent experiments), and *t* = 4, 6, 8, 12, 24, and 48 h postinoculation (average of *n* = 2 independent experiments per time point per growth condition), as described in “Materials and methods”. The *vertical dotted line* on the graph indicates that the *t* = 24 and 48 h data were obtained in a separate experimental run from the *t* = 4, 6, 8, and 12 h time points. *Error bars* = standard error of the mean (SEM)

**Fig. 2:**
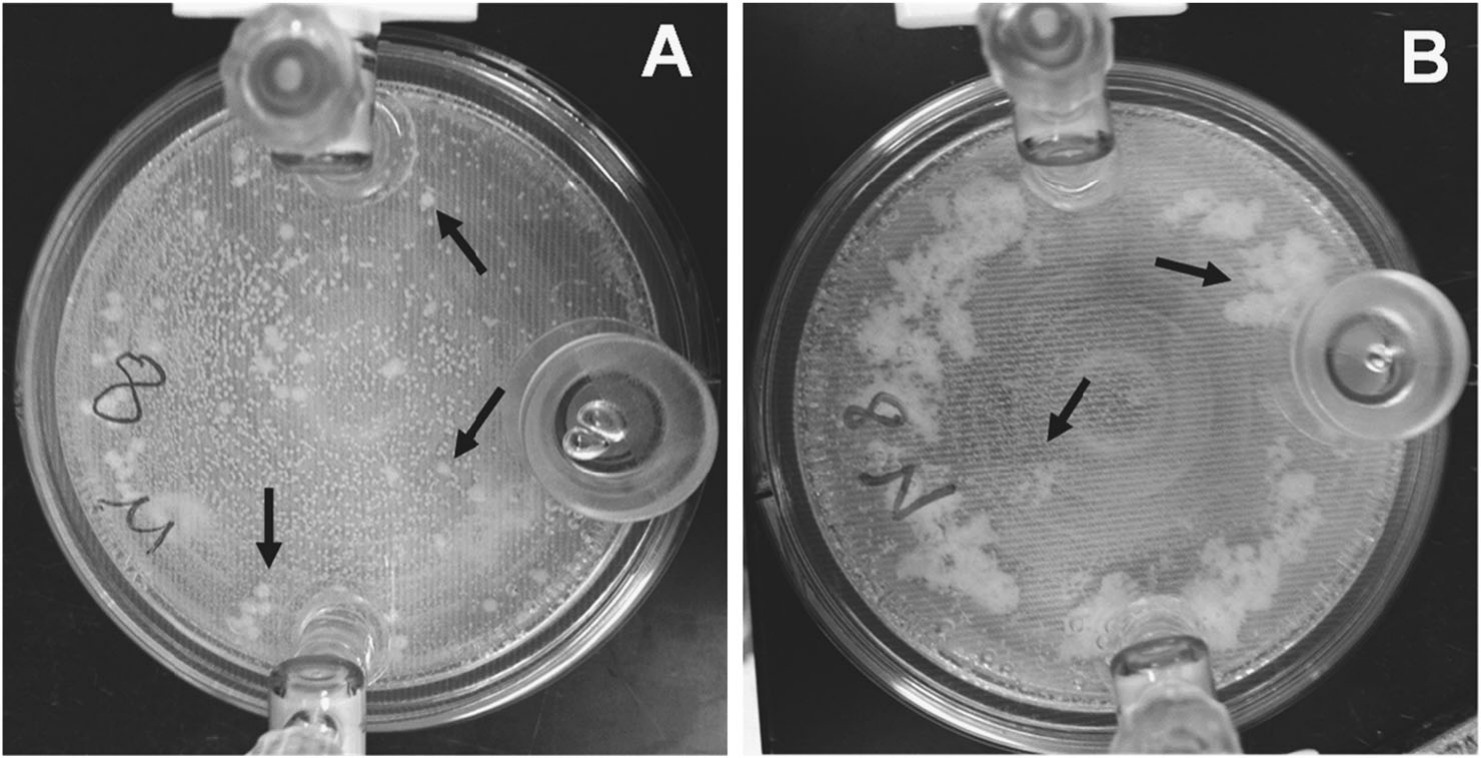
Representative *S. mutans* end-point HARV cultures grown for 8 h in biofilm media under simulated microgravity (**a**) and normal gravity (**b**) conditions. Note the structural differences of the self-aggregating bacterial cells under each condition (indicated by *arrows*)

Monitoring of cell growth by CFU/ml in the continuous growth curve experiments described above required constant disruption of the HARV cultures, making it difficult to assess potential macroscopic differences in cell aggregation in the simulated microgravity and normal gravity conditions. Therefore, these growth curves were also performed using an “end-point” approach, whereby simulated microgravity and normal gravity HARV cultures (*n* = 2 independent experiments per time point per growth condition) were grown undisturbed in replicate HARV vessels for *t* = 4, 6, 8, 12, 24, and 48 h (Fig. 1). Using this end-point approach, no significant differences in CFU/ml were observed between the simulated microgravity and normal gravity HARV cultures at the assessed time points (Fig. 1), and the initial drop in CFU/ml observed early in the growth curves of the continuous cultures (Supplemental Fig. S1) was not observed in these end-point cultures. As well, both the simulated microgravity and normal gravity cultures underwent the same pattern of growth when monitored with this end-point approach: Log phase growth occurred between 0 and 6 h, and both cultures were in early stationary phase at 8 h growth, followed by entry into late stationary phase/death phase between 24 and 48 h growth. Interestingly, differences in macroscopic cell aggregation were consistently observed between the simulated microgravity and normal gravity HARV conditions when using the end-point growth analysis. Specifically, the simulated microgravity condition tended to form round/compact cellular structures, whereas the aggregates in the normal gravity growth condition tended to form less compact structures (Fig. 2).

### Metabolomics analysis of “end-point” HARV cultures

**Fig. 3:**
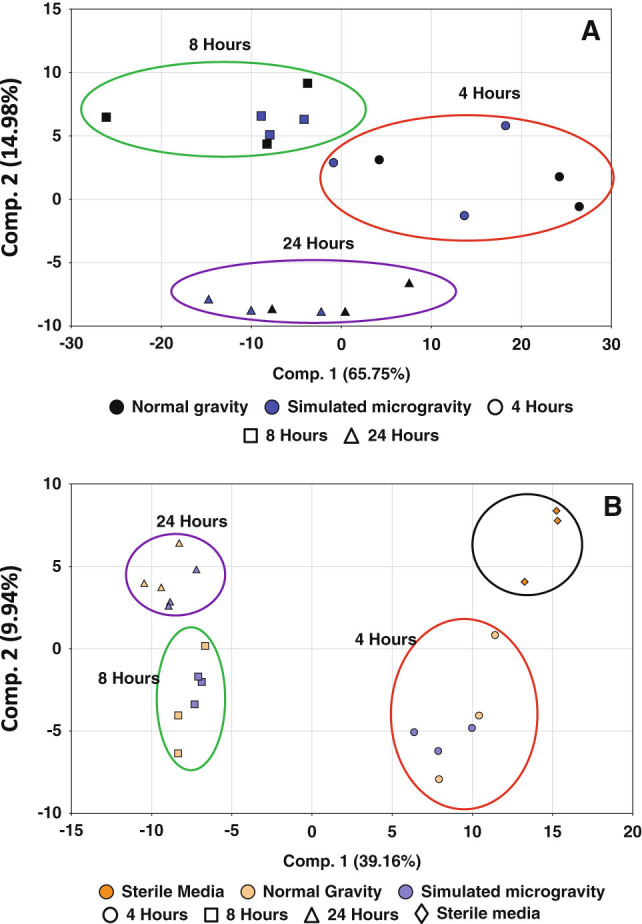
Principle component analysis (PCA) of cellular (**a**) and supernatant (**b**) metabolites. Values on *X* and *Y* axis labels = % of the total eigen values

To monitor potential differences in the metabolome of simulated microgravity and normal gravity HARV cultures, cell pellets and culture supernatants were harvested from a second end-point HARV culture experiment (*n* = 3 independent experiments per time point per growth condition) at *t* = 4, *t* = 8, and *t* = 24 h samples, corresponding to log-phase, early stationary phase, and late stationary/death phase, respectively **(**Supplemental Fig. S2 **)**. Given that macroscopic differences in the self-aggregating clusters of *S. mutans* cells were consistently observed at the *t* = 8 h time point (Fig. 2), RNA-seq analysis was also performed on the *t* = 8 h samples. Principle component analysis (PCA) of metabolomics data generated from *t* = 4, 8, and 24 h HARV cultures (Supplemental Files S2 and S3) revealed a strong relationship between the time of growth (*t* = 4, 8, and 24 h) and metabolite profile for each of the simulated microgravity and normal gravity cultures (Fig. 3). However, a much weaker “time-treatment” relationship existed when comparing the metabolite profile between simulated microgravity vs. normal gravity cultures at each time point. Welch’s two sample *t* tests revealed 8, 14, and 10 statistically significant differences (*p* ≤ 0.05) in cellular metabolite production between cells grown under normal gravity and microgravity conditions at the 4, 8, and 24 h time points, respectively (Supplemental File S2). Likewise, 21, 21, and 23 statistically significant differences were observed between the corresponding culture supernatant samples at these time points (Supplemental File S3). Keeping in mind the caveat that the number of significant differences at each time point were close to that expected by random chance alone (5% error rate), the significant changes of cell-associated and supernatant metabolites of the 8 h HARV cultures are summarized in Table 1, and their potential relationship to the corresponding 8 h RNA-seq data are discussed below.

**Table 1:** List of cellular and supernatant metabolites that displayed a significant (*p* < 0.05) time/growth condition interaction at *t* = 8 h HARV growth

Metabolite	Fold- increase/decrease (simulated microgravity/normal gravity)	*p*-value (Welch’s two sample *t* test)
Cellular (14 total)		
Serine	2.16	0.04
O-acetylserine	2.64	0.02
Phenylpyruvate	2.31	0.04
4-Hydroxyphenylpyruvate	3.25	0.004
4-Methyl-2-oxopentanoate	2.91	0.008
3-Methyl-2-oxovalerate	2.95	0.006
Argininosuccinate	4.21	0.01
Pyruvate	2.42	0.05
Alpha-ketoglutarate	2.95	0.04
Acetoacetate	4.54	0.001
4-Hydroxy-2-oxoglutaric acid	2.28	0.03
Adenine	1.82	0.04
Uridine monophosphate (5′ or 3′)	2.49	0.04
Uracil	3.76	0.001
Supernatant (21 total)		
Glycine	0.63	0.003
Alanine	0.76	0.02
*N*-acetylalanine	0.56	<0.001
Aspartate	0.75	0.01
Pyroglutamine	0.67	0.003
Citramalate	0.47	<0.001
4-Guanidinobutanoate	1.43	0.006
Gamma-glutamylvaline	1.39	0.02
Alanylglycine	0.76	0.02
Alanylisoleucine	1.17	0.03
Asparagylisoleucine	0.66	0.02
Cyclo(Leu-Pro)	0.91	0.03
Glycylphenylalanine	0.84	0.01
Leucylglycine	0.84	0.03
Threonylisoleucine	1.37	0.02
Glucose	0.12	0.006
Fructose	0.51	0.02
Guanine	1.66	0.001
Nicotinate	0.32	<0.001
3,4-Dihydroxybenzoate	1.59	0.01
1-Kestose	1.49	0.001

### RNA-seq analysis

**Fig. 4:**
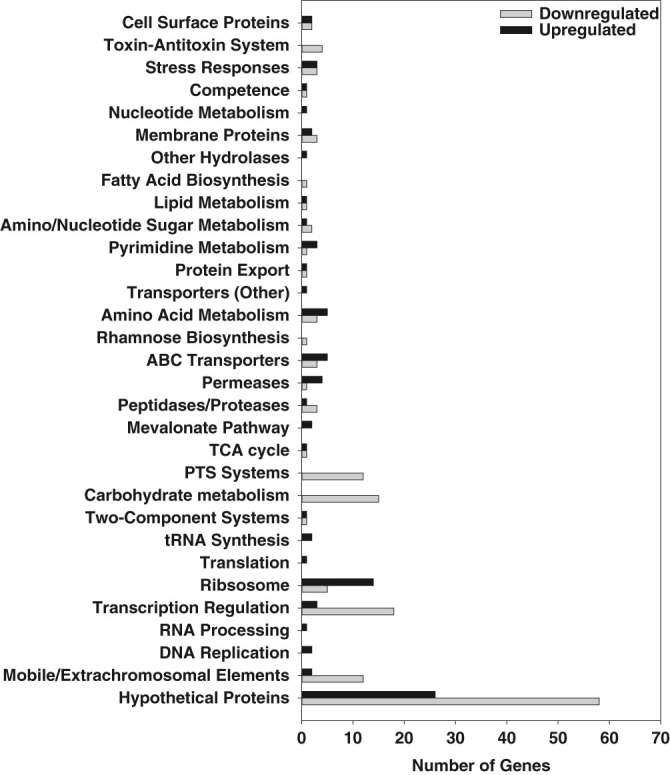
Distribution of gene functional categories differentially expressed during normal gravity growth relative to simulated microgravity. RNA isolated from 8 h normal gravity and simulated microgravity *S. mutans* HARV cultures (*n* = 3 independent experiments per growth condition) was subjected to RNA-seq transcriptome profiling as described in “Materials and methods”. Differential expression and statistical analysis was carried out using a cutoff FDR-adjusted *p* value of 0.05. Of this dataset, those genes with a log_2_ (fold-change) value of at least ±1.0 were grouped by functional classification based on DAVID gene functional categorization analysis, NCBI gene annotations and KEGG pathway analysis. Total number of up-regulated genes (log_2_ fold-change ≥1.0, *black bars*) = 94, total number of down-regulated genes (log_2_ fold-change ≤ −1.0, *gray bars*) = 153

Because macroscopic structural differences between the *S. mutans* simulated microgravity and normal gravity HARV cultures were consistently observed at 8 h growth (early stationary phase), we chose to perform RNA-seq analysis on *n* = 3 independent biological samples per growth condition (simulated microgravity and normal gravity) isolated from this time point. Compared to the metabolomics data, differential expression analysis of the 8 h RNA-seq data revealed a large number of statistically significant gene expression changes in normal gravity relative to simulated microgravity growth (Supplemental File S4). Specifically, 94 genes were up-regulated (log_2_ fold-change ≥1.0) and 153 genes were down-regulated (log_2_ fold-change ≤−1.0) in normal gravity cultures relative to simulated microgravity cultures. These patterns in gene expression were confirmed by qPCR for a subset of genes (Supplemental Table S1). Functional classification (Fig. 4) revealed that expression of a large number of genes associated with transposon sequences, as well as several genes encoding predicted phage proteins, were down-regulated in normal gravity cultures relative to simulated microgravity growth. A second trend of interest was the significant down-regulated expression of genes involved in carbohydrate metabolism, including various phosphotransferase (PTS) systems, the carbon catabolite protein regulator *ccpA*, and the redox sensitive transcriptional regulator *rex*, in the 8 h normal gravity cultures relative to the simulated microgravity cultures (Fig. 4 and Supplemental File S4). Increased expression of genes encoding ribosomal proteins, tRNAs and other aspects of protein translation was also observed in the normal gravity cultures compared to simulated microgravity growth, whereas expression of a large number of genes encoding transcriptional regulators was decreased in the normal gravity cultures (Fig. 4). As well, altered expression of stress genes was also observed in the normal gravity HARV cultures, including decreased expression of the toxin–antitoxin (TA) systems *mazEF* (SMU_172/173) and *relBE* (SMU_895/896), decreased expression of the holin-like *lrgA*, decreased expression of the peroxide resistance gene *dpr*, and increased expression of genes encoding glutathione S-transferase (SMU_1296) and glutaredoxin (SMU_669c) (Fig. 4).

DAVID bioinformatics resource^22, 23^ was used to further probe the RNA-seq dataset for functional annotation groups that were enriched in the genes that displayed a log_2_ fold change expression of at least ±1.0 in the normal gravity vs. simulated microgravity HARV cultures (Supplemental Table S2). This analysis revealed the presence of four significant (enrichment scores ≥1.3) functional annotation clusters in the down-regulated normal gravity genes, which included genes involved in carbohydrate metabolism and transport (cluster I), transcriptional regulators (clusters II and III), and carbohydrate transporters/other membrane proteins (cluster IV). As well, functional annotation clusters involved in arginine metabolism (cluster V) and nucleoside/tRNA metabolism (cluster VI) were significantly enriched (enrichment scores ≥1.3) in the up-regulated normal gravity genes. To identify metabolic pathways potentially altered under the simulated microgravity growth condition, all statistically-significant changes in 8 h growth gene expression from the RNA-seq differential expression analysis with a minimum log_2_ fold-change of at least ±0.6 were also analyzed using the bioinformatics program Metacyc.^24^. The top metabolic pathways identified by this analysis were mixed acid fermentation (seven genes), arginine biosynthesis (six genes), and pyrimidine deoxyribonucleotide biosynthesis (five genes) (Supplemental Fig. S3). Specifically, genes involved in the conversion of acetyl-CoA to 2-oxoglutarate (a TCA cycle intermediate), arginine biosynthesis, and dCDP/dTMP biosynthesis were upregulated in the normal gravity HARV cultures, whereas genes involved in ethanol and acetate fermentation were down-regulated in normal gravity HARV cultures. Interestingly, when comparing these metabolic pathways to statistically-significant changes in cellular metabolites at 8 h growth (Table 1), several matched to the corresponding metabolic pathway changes (Supplemental Fig. S3) identified in the RNA-seq data by Metacyc, including 2-oxogluarate (alpha-ketoglutarate; mixed acid fermentation), pyruvate (mixed acid fermentation), argininosuccinate (arginine biosynthesis), uridine monophosphate and uracil (pyrmidine deoxynucleotide de novo biosynthesis). The apparent increased sensitivity of the RNA-seq data in detecting more changes in *S. mutans* metabolism relative to the corresponding metabolomics data may reflect a suboptimal component of the metabolomics sample collection method and/or processing time.

### Assessment of oxidative stress and competence phenotypes

**Fig. 5:**
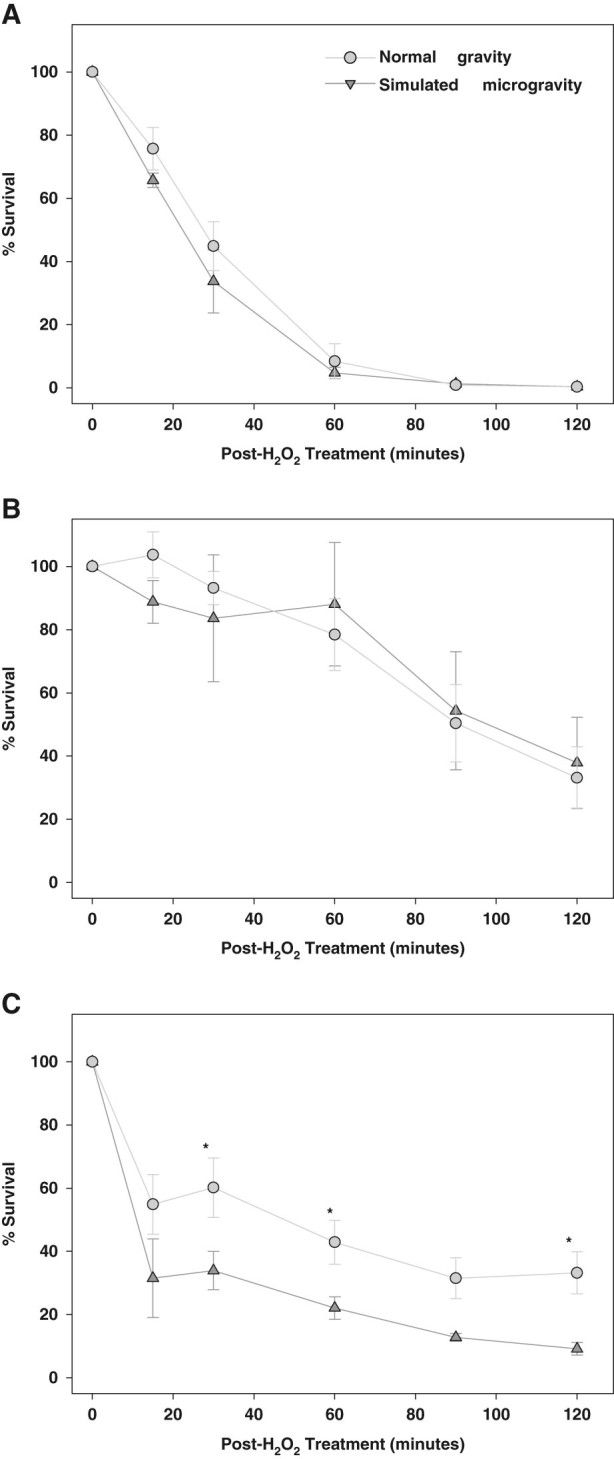
Percent survival of *S. mutans* post-treatment with 5 mM H_2_O_2_. Cells from *t* = 4 (**a**), *t* = 8 (**b**), or *t* = 16 (**c**) hour simulated microgravity (*triangles*) and normal gravity (*circles*) HARV cultures (*n* = 3 independent experiments per time point per growth condition) were harvested by centrifugation and resuspended in sterile HBSS. Samples were withdrawn to determine initial CFU/ml (“*t* = 0”), and then H_2_O_2_ was added to a final concentration of 5 mM. CFU/ml of each cell suspension was sampled at 20, 40, 60, 90, and 120 min post-H_2_O_2_ addition, and CFU/ml were determined. % Survival was calculated by 100 × [(*t* = final CFU/ml)/(*t* = 0 CFU/ml)]. *Error bars* = SEM. *Asterisk* denotes statistical significance, one-tailed *t* test (*p* < 0.05)

*S. mutans* encounters significant oxidative stress in the oral cavity in the form of H_2_O_2_ production by competing non-cariogenic oral streptococci,^25,26^ as well as from dental care products (toothpastes, mouthwash) that contain H_2_O_2_. Furthermore, expression of several oxidative stress-related genes (*dpr*, *rex,* SMU_1296, SMU_669c) was altered in the 8 h simulated microgravity HARV cultures. Therefore, the ability of *S. mutans* to survive exogenous H_2_O_2_ treatment was compared in cells isolated from exponential (*t* = 4), early-stationary (*t* = 8) and late stationary (*t* = 16) simulated microgravity and normal gravity HARV cultures (Fig. 5). Although no significant differences in oxidative stress resistance were observed between normal gravity and simulated microgravity cells isolated from 4 and 8 h growth, the H_2_O_2_-treated 4 h cells (Fig. 5a) displayed a greater overall loss of viability (~1% survival after 90 min of treatment) compared to the 8 h cells (Fig. 5b; ~50% survival after 90 min of treatment). Interestingly, when this experiment was repeated on cells from normal gravity and simulated microgravity cultures harvested at 16 h growth (Fig. 5c), the simulated microgravity cells displayed significantly (*P* < 0.05, one-tailed *T* test) increased H_2_O_2_ killing (~22% survival after 60 min treatment) compared to the normal gravity cells (~43% survival after 60 min treatment).

**Fig. 6:**
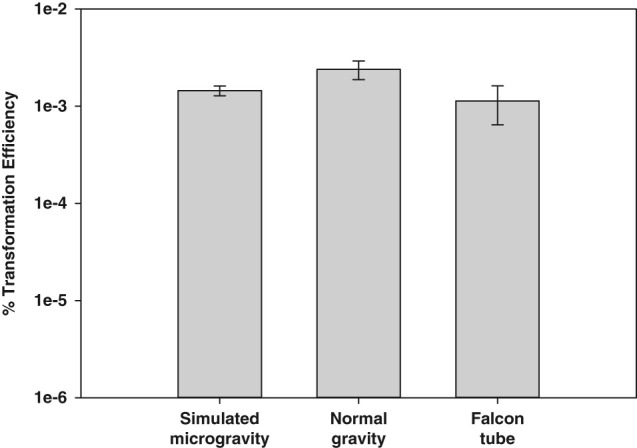
*S. mutans* competence phenotype assessed in simulated microgravity and normal gravity HARV cultures. Simulated microgravity and normal gravity HARV cultures, as well as a parallel static Falcon tube control culture, were each grown for 1.5 h in biofilm media prior to addition of 2 µg pOri23 plasmid DNA. All three cultures were then grown for 2.5 more hours followed by harvest and serial dilution plating on BHI agar ± 10 µg/ml erythromycin (selective antibiotic to monitor plasmid transformation). Transformation efficiency was calculated as the percentage of transformants (CFU/ml on BHI + erythromycin) among total viable cells (CFU/ml on BHI). *Data* represent the average of *n* = 3 independent experiments. *Error bars* = SEM

Because previous *S. mutans* studies have implicated a complex link between oxidative stress, biofilm formation, and natural competence [reviewed in^27^], we were curious if competence was also subject to regulation by the low-shear modeled microgravity ﻿(LSMMG) environment. Therefore early-exponential phase (growth phase at which *S. mutans* is most competent during standard in vitro growth) simulated microgravity and normal gravity HARV cultures were tested for their ability to uptake plasmid DNA. However, transformation efficiency (as measured by uptake of a plasmid conferring erythromycin resistance) was nearly identical between simulated microgravity and normal gravity HARV cultures (Fig. 6). The transformation efficiency was also not affected by HARV growth itself, as the HARV transformation efficiencies were similar to those measured in a parallel non-rotating culture of *S. mutans* (Fig. 6).

## Discussion

Although the effects of both spaceflight and microgravity analog growth on virulence-related phenotypes of bacterial pathogens such as *Salmonella enterica* serovar Typhimurium, *Escherichia coli*, *Pseudomonas aeruginosa*, and *Staphylococcus aureus* have been well-described,^28–38^ comparatively little research has been dedicated to studying the specific effects of space flight and simulated microgravity on virulence attributes of oral bacteria such as *S. mutans*. In the Skylab missions conducted in the 1970s, the effects of space flight and diet on the oral cavity and oral microbiota of astronauts were documented using culture-dependent methods. These studies demonstrated that increased counts of specific oral bacteria (i.e. *Bacteriodes*, *Veillonella*, *Fusobacterium*, *Neisseria*, *S. sanguinus*, and *S. mutans*) were recovered from the astronauts in response to space flight.^16^ Furthermore, the astronauts in these studies exhibited decreased salivary lysozyme and increased dental calculus and gingival inflammation.^16^ More recently, the effect of simulated microgravity on the physiology and biofilm structure of *S. mutans* was also assessed using a superconducting magnet capable of generating a large gradient high magnetic field (LG-HMF),^39^ which mimics the space-like gravity environment by achieving stable levitation of diamagnetic materials such as biological cells and tissues.^40^ The results of this study suggested that growth of *S. mutans* under simulated microgravity enhanced its acid tolerance, altered its biofilm architecture, and increased its proportion within dual-species biofilms co-cultured with *S. sanguinis*, an H_2_O_2_-producing early colonizer of dental plaque biofilm.^39^ Collectively, these previous studies suggest that both space flight and the microgravity analog environment have the ability to alter the cariogenic potential of *S. mutans*.

In our current work, HARVs rotated on an axis perpendicular to the Earth’s gravitational vector were used to simulate a low-shear modeled microgravity (LSMMG) growth environment. This ground-based model of microgravity has been used to study the response of a variety of microorganisms to “weightlessness” that would be encountered during space flight [reviewed in^19, 41, 42^]. However, it is important to keep in mind that the *S. mutans* LSMMG-associated metabolic and physiological changes (i.e. alterations in cell aggregation, transcriptome, and sensitivity to oxidative stress) presented here could represent the bacteria’s response to either simulated microgravity and/or low-fluid shear (<1 dyn/cm^2^) specific environmental signals, the latter of which may be relevant to the fluid shear of saliva flow (<0.8 dyn/cm^2^) experienced by *S. mutans* in the oral cavity.^19,21^ Therefore, the data obtained in this study have the potential to shed light on *S. mutans* physiology and virulence properties relevant to environmental signals experienced in the host, and will hopefully provide a springboard for planning future space flight studies of oral bacteria. It is also important to note that all of the *S. mutans* experiments presented in this study were conducted in semi-defined culture medium containing 11 mM glucose and 10 mM sucrose, previously shown to promote in vitro *S. mutans* biofilm formation.^43^ The addition of sucrose, a host dietary sugar to which *S. mutans* is exposed to in the oral cavity, promotes production of extracellular glucan polymers by *S. mutans*, important biofilm matrix components that promote intracellular adhesion.^7^ In reality, the in vivo plaque biofilm environment of *S. mutans* is much more complex, including stress conditions such as interspecies competition for nutrients and space within the biofilm, as well as alternating periods of “feast and famine” with respect to dietary carbohydrate consumption. Therefore, the growth condition used in this study should be considered a simple representation of caries-promoting “feast” conditions experienced by *S. mutans* in the oral cavity. With these caveats in mind, the following discussion points represent interesting patterns/conclusions from the data obtained in this study:

1. *S. mutans* displays an altered early stationary-phase metabolism under simulated microgravity conditions: As indicated in the “Results” section and Fig. 1, *S. mutans* “end-point” simulated microgravity and normal gravity cultures appear to enter stationary phase at the same point in growth (6 h) compared to normal gravity cultures, and there were no apparent differences in CFU/ml of each culture at each time point. This being said, the 8 h (early stationary phase) metabolomics and RNA-seq data suggest that *S. mutans* simulated microgravity metabolism at this point is altered relative to normal gravity growth. For example, simulated microgravity cultures experienced a ten-fold decrease in supernatant glucose levels (Table 1 and Supplemental File S3) at 8 h growth relative to the normal gravity cultures. Furthermore, expression of PTS system genes involved in transport of carbohydrates (glucose, mannitol, trehalose, mannose and cellobiose) was significantly upregulated during simulated microgravity growth, whereas expression of genes encoding ribosomal proteins, tRNAs, and other aspects of protein translation was decreased in the microgravity cultures at this time point (Fig. 4 and Supplemental File S4 ). Bioinformatics analysis also revealed patterns of decreased arginine metabolism and decreased pyrimidine biosynthesis (Supplemental Table S2 and Supplemental Fig. S3) in the 8 h simulated microgravity cultures. Finally, expression of genes encoding the central metabolic transcriptional regulators CcpA and Rex were also both upregulated (2.2-fold and 2.8-fold, respectively) at 8 h growth in the simulated microgravity cultures. CcpA has been shown to regulate *S. mutans* catabolite repression and the ability to utilize non-preferred carbohydrates, acid tolerance, acid production, and biofilm formation,^44–46^ whereas Rex regulates expression of *S. mutans* genes involved with carbohydrate fermentation, biofilm formation, and oxidative stress resistance in response to cellular redox.^47,48^ Interestingly, a role for Rex in the transcriptional response of *Staphylococcus aureus* to LSMMG was also previously implicated by the altered expression of a number of Rex-regulated genes under this growth condition.^29^ Although great care was taken in each experiment to vigorously vortex all samples prior to and during serial dilution plating, we cannot exclude the possibility that sucrose-dependent cell clumping may have masked more apparent growth differences between the simulated microgravity and normal gravity end-point HARV cultures in these studies.

2. *S. mutans* late stationary-phase simulated microgravity cultures appear to be subject to increased stress: A striking phenotypic property observed in *S. mutans* simulated microgravity cultures was the greatly increased sensitivity of cells from late stationary phase (16 h) to H_2_O_2_ treatment (Fig. 5c) relative to cells from normal gravity cultures. However, no apparent differences in oxidative stress resistance were observed between the simulated microgravity and normal gravity cells harvested at earlier time points (log phase and early stationary phase, Fig. 5a, b, respectively). This result correlated with the LG-HMF *S. mutans* microgravity study by Cheng and co-workers,^39^ which found that mid-exponential phase *S. mutans* microgravity and normal gravity cells did not differ in their sensitivity to H_2_O_2_. In previous studies, late stationary-phase (20–24 h) cultures of *S. typhimurium* and *P. aeruginosa* were both shown to be more resistant to oxidative stress when grown in simulated microgravity,^30,49^ whereas *S. typhimurium* simulated microgravity cultures grown to an earlier phase of growth (10 h) displayed increased sensitivity to H_2_O_2_ treatment.^31^ Furthermore, the sensitivity of stationary phase simulated microgravity cultures of adherent-invasive *E. coli* to oxidative stress was shown to be strain-dependent.^33^ The susceptibility of *S. aureus* to oxidative stress when grown in LSMMG has also been previously investigated: compared to normal gravity growth, *S. aureus* cells isolated from simulated microgravity cultures displayed increased sensitivity to challenge with H_2_O_2_, which was demonstrated to be related to decreased carotenoid (yellow membrane pigment) production under this growth condition.^29^ These previously-published studies, in conjunction with the *S. mutans* results presented here, clearly demonstrate that the microgravity model used, growth phase at which oxidative stress is tested, and/or other inherent species and strain-dependent differences, make it difficult to discern broad conclusions regarding the effect of the LSMMG on oxidative stress resistance.

Although direct comparisons cannot be extrapolated due to time point differences between the 8 h RNA-seq data (Fig. 4 and Supplemental File S4) and 16 h oxidative stress data (Fig. 5c), it is interesting to note that the 8 h simulated microgravity cultures displayed increased expression of genes previously-shown to be involved in the response to oxidative stress in *S. mutans*. These included *lrgA*, which encodes a predicted holin-like protein,^50,51^ *dpr*, encoding an iron-binding protein that reduces the intracellular free iron pool during oxidative stress,^52,53^ and *rex*.^48^ As well, *S. mutans* simulated microgravity cultures displayed increased expression of genes encoding phosphotransacetylase (Pta; SMU_1043c) and two putative acetate kinase (Act) genes (*ackA* and SMU_1299c), involved in the conversion of acetyl-CoA to acetyl phosphate and acetyl phosphate to acetate, respectively.^54^ In *S. mutans*, aspects of the Pta-Ack pathway have been shown to influence the cellular response to oxidative stress resistance.^54^ These results suggest that the simulated microgravity HARV cultures may be experiencing an oxidative-like stress response in early stationary phase (8 h growth), and the altered expression of these oxidative-stress related genes and metabolic pathways is perhaps occurring in response to this stress. Further support for the idea that the *S. mutans* simulated microgravity cultures may be experiencing more inherent stress relative to the normal gravity cultures stems from the observation that TA system genes *mazEF* and *relBE* were significantly upregulated during simulated microgravity growth (Fig. 4 and Supplemental File S4). MazF and RelE have been shown in several bacteria to selectively inhibit translation by cleaving specific mRNA recognition sequences in response to nutritional stress [reviewed in^55,56^]. These data collectively suggest that *S. mutans* simulated microgravity HARV cultures display characteristics associated with stationary phase cellular stress. However, it is not clear from this data whether these cultures are experiencing overlapping simultaneous stresses (such as oxidative and starvation stress), or if the sum of these phenotype and gene expression changes represent a stress response specific to LSMMG.

3. *S. mutans* displays an altered cell aggregation phenotype under simulated microgravity conditions: Micro-organisms such as *Micrococcus luteus*,^57^ *E. coli*,^58^ *P. aeruginosa*,^34,35,59^ *S. aureus*,^29^ *S. typhimurium*,^36^ and *Candida albicans* ^60,61^ have all displayed increased biofilm growth when grown in either ground-based simulated and/or space flight microgravity conditions. In some instances, this biofilm increase was paralleled by documented increases in expression of biofilm-promoting regulatory genes or adhesion factors.^29,34–36,49,58,61^ Given these observations in other micro-organisms, we predicted that *S. mutans* would also display an increase in biofilm formation when grown in HARV microgravity cultures. Although biofilms attached to a solid surface were not assessed in these studies, macroscopic differences in cell–cell aggregation were typically observed in simulated microgravity compared to normal gravity HARV cultures at stationary phase (8 h growth; Fig. 2), suggesting that cell–cell adhesion and/or biofilm-like properties of this bacterium were altered in response to simulated microgravity growth. Although cell aggregation was observed in both the normal gravity and simulated microgravity HARV cultures grown in biofilm-promoting media containing sucrose, the simulated microgravity cell aggregates were more compact and more difficult to disrupt relative to the aggregates formed during normal gravity growth. Follow-up studies will be required to better characterize the structural properties of these cell aggregates (i.e. production of sucrose-dependent exopolysaccharide) and whether they are actually similar to biofilms that form on hydroxyapatite in vitro and/or the tooth surface in vivo. However, it is noteworthy that altered cell aggregation and/or “attachment-independent” phenotypes have also been observed in other biofilm-forming microbes in both LSMMG^29,34^ and during spaceflight.^61^

In summary, the results from our experiments suggest that *S. mutans* undergoes changes in metabolism, physiology, and global gene expression during stationary-phase simulated microgravity growth that impact cell aggregation and oxidative stress resistance. Defining the exact regulatory circuits and contributions of these differentially regulated genes to the ability of *S. mutans* to survive and adapt to LSMMG and spaceflight environments will help in the development of treatment and control strategies to combat the caries potential of this pathogen. Furthermore, these studies may provide insight into the response of *S. mutans* to low-fluid shear encountered in the oral cavity. Our future research efforts will focus on assessing *S. mutans* biofilm formation during simulated microgravity growth using culture conditions more closely mimicking the oral cavity (i.e., artificial saliva media and periodic pulsing of sucrose addition), as well as growth of *S. mutans* as part of a mixed-species biofilm consortium.

## Materials and methods

### Bacterial strain and growth conditions

*S. mutans* UA159^62^ was used for all of the experiments described below. All *S. mutans* cultures (including HARV vessels) were grown at 37 °C in a 5% CO_2_ incubator. For each experiment, *S. mutans* was freshly streaked from a frozen 30% (vol vol^−1^) glycerol stock onto Brain heart infusion (BHI) agar and grown for 48 h. A single colony was then inoculated into 30 ml BHI broth, and grown as a static culture for 16–18 h prior to inoculation of HARVs. Disposable vessel rotary cell culture systems (Synthecon) with vertical and horizontal 4-station rotator bases were used to simulate microgravity (HARV rotated on axis perpendicular to gravitational vector) and “normal” (1 × g) gravity (HARV rotated on axis parallel to gravitational vector), respectively (Supplemental Figure S4). All HARV manipulations were performed under a Class II A2 biosafety cabinet (Labconco). HARV vessels were filled with sterile media according to manufacturer’s recommendations, and incubated at room temperature for 18 h prior to each experiment. This media was then aseptically removed, and 20× dilute *S. mutans* overnight culture (approximate OD_600_ = 0.06/ml) in room-temperature semi-defined biofilm media (BM)^43^ containing 11 mM glucose and 10 mM sucrose, was used to fill each HARV. Prior to HARV rotation and incubation, sterile 5-ml luer lock syringes were used to remove air bubbles according to manufacturer’s protocols.

### Continuous HARV growth curves

For each experiment (*n* = 3 independent experiments per growth condition), overnight *S. mutans* cultures were used to inoculate 120 ml of sterile BM containing 11 mM glucose and 10 mM sucrose. This inoculum was mixed well by vigorous swirling and vortexing, and 1 ml withdrawn to measure the time-of-inoculation (*t* = 0) CFU/ml. The remaining inoculum volume was then subjected again to vigorous swirling and vortexing and immediately used to fill 2 × 50 ml disposable HARVs as described above. Each HARV was rotated either vertically (“simulated microgravity”) or horizontally (“normal gravity”) at 37 °C, 5% CO_2_ for 24 h. HARVs were rotated at 30 RPM for the first 8 h of growth, and the rotation speed of each reactor was increased to 32 RPM after 8 h of growth to ensure that *S. mutans* cell aggregates did not settle to the bottom of the simulated microgravity HARV. At 2, 4, 6, 8, 12, and 24 h postinoculation, each HARV was removed from the incubator, vigorously agitated to remove adherent cell clumps from the walls and membrane of the HARV reactor and evenly dispersing cell clumps, and 1 ml culture was immediately removed for CFU/ml assessment. The same volume of sterile media was then added back to each HARV and air bubbles removed as described above prior to re-initiation of simulated microgravity or normal gravity HARV growth. For CFU/ml quantification, each 1 ml sample was vortexed vigorously for at least 30 s to resuspend cell clumps, followed immediately by serial dilution (each tube vortexed vigorously for at least 15 s between each dilution) and CFU/ml determination using the track plating method.^63^

### HARV “end-point” growth curves

Overnight *S. mutans* cultures were used to inoculate 500 ml sterile BM containing 11 mM glucose and 10 mM sucrose to an approximate OD_600_ = 0.06/ml as described above. This inoculum was mixed well by vigorous swirling and vortexing, and 1 ml was withdrawn to measure the *t* = 0 OD_600_, pH, and CFU/ml. The remaining inoculum volume was then immediately used to fill 8 × 50 ml disposable HARVs (inoculum volume was subjected to vigorous swirling and vortexing before addition to each HARV). For end-point growth curve analysis at *t* = 4, 6, 8, and 12 h (*n* = 2 independent experiments total per time point per growth condition), four simulated microgravity and four normal gravity HARV cultures were grown in parallel at 37 °C, 5% CO_2_. HARVs were rotated at 30 RPM for the first 8 h of growth, and the rotation speed of each reactor was increased to 32 RPM after 8 h of growth to ensure that *S. mutans* cell aggregates did not settle to the bottom of the simulated microgravity cultures. At each time point, one simulated microgravity and one normal gravity HARV were each harvested as follows: 25 ml of HARV culture was transferred to a sterile 50 ml Falcon tube. The HARV was then closed and shaken vigorously for approximately 5 s (until there were no remaining visible clumps of bacteria stuck to the inside walls and membrane of the HARV). The reactor was then immediately uncapped and the remaining culture transferred to the 50 ml Falcon tube, which was vortexed at high speed for 30 s prior to removing 100 µl for CFU counts. The tube was vortexed again for 10 s, and 900 µl immediately removed for measuring OD_600_ and pH readings. OD_600_ readings were obtained using a Genesys 10 Bio spectrophotometer (Thermo Scientific), culture pH measured with colorpHast indicator strips (EMD), and CFU/ml calculated by serial dilution plating as described above. For the end-point growth curve analysis at *t* = 24 and *t* = 48 h, a separate experiment (*n* = 2 biological replicates for each time point) was performed as described above.

### HARV end-point growth for metabolomic and RNA-seq analyses

Overnight *S. mutans* cultures were used to inoculate 350 ml sterile BM containing 11 mM glucose and 10 mM sucrose to an approximate OD_600_ = 0.06/ml as described above. This inoculum was mixed well by vigorous swirling and vortexing, and 1 ml was withdrawn to measure the *t* = 0 OD_600_, pH, and CFU/ml. The remaining inoculum volume was then immediately used to fill 6 × 50 ml disposable HARVs (inoculum volume was subjected to vigorous swirling and vortexing before addition to each HARV). A sterile media control was also collected at the beginning of each experiment and stored at −80 °C for metabolomics analysis (see below). For each experiment (*n* = 3 independent experiments total), three simulated microgravity and three normal gravity HARV cultures were grown in parallel at 37 °C, 5% CO_2_ as described above for the end-point growth curve analysis. For each experiment, one simulated microgravity and one normal gravity HARV were each harvested at *t* = 4, *t* = 8 h, and *t* = 24 h growth, as described above for the end-point growth curve analysis (for a total of *n* = 3 independent samples per time point per growth condition). HARVs were photographed prior to culture disruption and harvest to document macroscopic growth observations. After harvesting, 34 ml of each *t* = 4, 8, and 24 h HARV culture was centrifuged at 3000 RPM, 4 °C for 20 min and immediately placed on ice. 1 ml of culture supernatant was removed from each centrifuged sample, transferred to 1.5 ml tubes, and immediately frozen at −80 °C. The remaining culture supernatants were decanted and cell pellets immediately frozen at −80 °C. For RNA-seq samples (*t* = 8 h only), a 10-ml aliquot of each HARV culture was also centrifuged at 3000 RPM, 4 °C for 20 min and immediately placed on ice. Culture supernatants were decanted and 1 ml RNAlater (Ambion) was added to each cell pellet prior to immediate storage at −80 °C.

### Metabolite extraction and metabolomics analysis

Extraction of metabolites (*n* = 3 independent biological samples per time point and growth condition) and all metabolomics analyses (including statistics) were performed by Metabolon, Inc. (Durham, NC) following previously-published methods.^64–68^ Details specific to this experiment can be found in Supplemental File S1 (Supplemental Materials and Methods).

### RNA isolation and RNA-seq analysis

RNA was isolated from *t* = 8 h cell pellets stored at −80 °C in RNAlater (collected from *n* = 3 independent experiments each for simulated microgravity and normal gravity HARV cultures) with the RNeasy Kit (Qiagen) and FASTPREP lysing matrix B tubes (MP Biomedical) using previously-described methods.^50,69^ Each RNA sample was then subjected to a second DNAse treatment using the TURBO DNA-free™ Kit (Thermo Fisher Scientific) per the manufacturer’s protocols. Lack of contaminating genomic DNA in each RNA sample was then determined using quantitative real-time PCR (i.e. no cDNA synthesis step) and *S. mutans*-specific primers (1547-F/R; Supplemental Table S3) prior to sending ≥5 µg of each RNA sample to Seqwright Genomic Services (Houston, TX), who performed all steps of the RNA-seq workflow, data curation, differential expression, and corresponding statistical analysis. In brief, each RNA sample was subjected to an mRNA enrichment step and generation of a library of cDNA template molecules of known strand origin using the TruSeq Total mRNA Sample Preparation Kit (Illumina). Sequencing was performed using the Illumina HiSeq 2500 platform, generating 2 × 100 bp read lengths for a total of 2 × 10 M reads per sample. Illumina CASAVA software was used to create genomic builds and count reads, whereas TopHat-2.0.8b and Cufflinks-2.0.1^70^ were used to align reads to the *S. mutans* UA159 genome (Genbank accession # NC_004350.2). ﻿All RNA-seq data have been deposited to NCBI's Gene Expression Omnibus and are accessible through GEO Series accession number GSE90166.﻿

### Hydrogen peroxide (H_2_O_2_) assay

For each experiment (*n* = 3 independent biological samples each for simulated microgravity and normal gravity HARV cultures), two *S. mutans* 10-ml HARV cultures were inoculated as described above, and each was grown for either 4, 8, or 16 h. For the 4 and 8 h experiments, the HARV rotation speed was 30 RPM. For the 16 h experiment, the HARV rotation speed was 30 RPM for the first 8 h of growth, and was then increased to 35 RPM to counteract sedimentation of cellular aggregates. *S. mutans* cultures were then harvested from each HARV vessel, transferred to a 50 ml Falcon tube, and centrifuged for 10 min at 4000 RPM. Cell pellets were resuspended in 5 ml sterile 1× Hanks Buffered Salt Solution (HBSS), and 100 µl was withdrawn from each tube and subjected to serial dilution plating as described above to enumerate CFU/ml of each cell suspension at “*t* = 0” (prior to H_2_O_2_ addition). H_2_O_2_ was added to each remaining 4.9 ml cell suspension to a final concentration of 5 mM, and incubated at 37 °C, 5% CO_2_. Samples of each cell suspension were removed at 15, 30, 45, 60, and 120 min post-H_2_O_2_ addition to enumerate CFU/ml, and % survival for each sample was calculated by 100× (*t* = × CFU/ml/*t* = 0 CFU/ml).

### Competence assay

The ability of *S. mutans* to take up exogenous DNA was assessed in microgravity and normal gravity HARV cultures based on previously-described methods.^50,71^ For each experiment (*n* = 3 independent biological samples each for simulated microgravity and normal gravity HARV cultures), *S. mutans* 10-ml micro-gravity and normal gravity HARV cultures were grown in BM containing 11 mM glucose and 10 mM sucrose for 90 min [corresponding to 2.3 × 10^6^ CFU/ml (±3.0 × 10^5^ SEM) for normal gravity and 3.1 × 10^6^ CFU/ml (±1.5 × 10^5^ SEM) for simulated microgravity] at 30 RPM. HARVS were removed from rotator bases and 2 µg of unmethylated pOri23 plasmid DNA was added to each reactor. Microgravity and normal gravity HARVs were then incubated for 2.5 h at 30 RPM prior to serial dilution and plating of each culture on BHI agar ±10 µg/ml erythromycin (Erm; selectable antibiotic for pOri23). CFU/ml were determined after 48 h plate growth, and transformation efficiencies were calculated as the percentage of transformants (BHI-Erm^10^ CFU/ml) among the total viable cells (BHI CFU/ml). For each experiment, a non-rotating control culture (15-ml Falcon tube containing 15 ml of *S. mutans* culture) was grown in parallel and assessed for competence as described above.

### Bioinformatics

The DAVID Bioinformatics Resource^22,23^ was used to aid in functional categorization of the RNA-seq differential expression data and to perform functional annotation clustering. Only genes with statistically-significant log2-fold changes of at least ±1.0 were included in these analyses. The lowest classification stringency was used for functional categorization. For functional annotation clustering analysis, the default analysis parameters were used: Medium classification stringency, similarity threshold = 0.5, enrichment threshold (EASE score) = 1.0. As recommended previously,^22^ an enrichment score cutoff of ≥1.3 was applied to the functional annotation clustering analysis. For identifying trends in metabolic pathway changes, all statistically-significant RNA-seq differential expression data with a log_2_-fold change of at least ±0.6 were also analyzed using Metacyc.^24^

### Statistics

For metabolomics, statistical analyses were performed in ArrayStudio on log transformed data. For those analyses not standard in ArrayStudio, the programs R (http://cran.r-project.org/) or JMP (SAS, http://www.jmp.com) were used as previously described.^72^ Two-way ANOVA was used to compare the effects of treatment (simulated microgravity vs. normal gravity growth) and growth time (*t* = 4, 8, and 24 h) on metabolite differences. Multiple comparisons were accounted for with the false discovery rate (FDR) method, and each FDR was estimated by *q*-values. PCA was also applied to the metabolomics data. For RNA-seq, differential expression analysis was performed by Seqwright Genomic Services using FPKM (fragments per kilobase per million mapped reads) values and fold-change was expressed as log_2_ (FPKM normal gravity/FPKM simulated microgravity). For all other data, statistical analyses were performed using Sigmaplot version 12.5 (Build 12.5.0.38, Systat Software, Inc.). Data was tested for normality and equal variance, followed by a *T*-test or Rank-sum test, as appropriate.

## Electronic supplementary material

Supplementary Tables

Supplementary Figures

Supplementary File S1

Supplementary File S2

Supplementary File S3

Supplementary File S4
