# Supplementary material for: Investigation of simulated microgravity effects on Streptococcus mutans physiology and global gene expression
Source: NPJ Microgravity. 2017 Jan 12;3:4. doi: 10.1038/s41526-016-0006-4 (PMC5460135; doi:10.1038/s41526-016-0006-4)
Supplement: Supplementary file 1 — Supplementary Tables [file 41526_2016_6_MOESM1_ESM.docx]

**Table S1. Comparison of log_2_ fold-change expression values by RNA-seq and qPCR for select differentially expressed genes under normal gravity growth relative to microgravity growth.**

|  |  | Normal gravity/  Microgravity | |
| --- | --- | --- | --- |
| Gene name | **Predicted function** | **RNA-seq log_2_**  **fold-change** | **qPCR log_2_ fold-change** |
| SMU_2003a | 50S ribosomal protein L36 | 1.41 | 1.62 |
| SMU_669c | putative glutaredoxin | 1.28 | 1.87 |
| SMU_917c | 6-pyruvoyl tetrahydrobiopterin synthase | 2.25 | 2.20 |
| SMU_1296 | putative glutathione S-transferase | 1.28 | 1.45 |
| *mazF* | ppGpp-regulated growth inhibitor (ChpA/MazF) | -2.60 | -2.87 |
| *wapA* | cell wall-associated protein | -2.08 | -1.45008 |
| SMU_1053 | rex homolog | -1.49 | -1.13289 |
| SMU_1291c | putative chorismate mutase | -1.45 | -1.21424 |
| *ptcB* | PTS system, cellobiose-specific IIB component | -2.36 | -1.60823 |
| *lrgA* | holin-like membrane protein | -2.37 | -2.46594 |

**Table S2. DAVID functional annotation clustering analysis of RNA-seq differentially expressed genes (normal gravity/simulated microgravity) with statistically-significant log_2_-fold changes of at least ± 1.0.**

|  | **Annotation Cluster** | **Enrichment Score** | **Categories/Terms** | **Genes** |
| --- | --- | --- | --- | --- |
| **DOWN-regulated**  **(Normal/**  **Simulated micro-G)** | **I**  **(11 genes)** | **2.06** | \| \| KEGG_PATHWAY \| smu00520:Amino sugar and nucleotide sugar metabolism \| \| --- \| --- \| \| KEGG_PATHWAY \| smu02060:Phosphotransferase system (PTS) \| \| INTERPRO \| IPR004720:Phosphotransferase system, sorbose subfamily IIB component \| \| KEGG_PATHWAY \| smu00051:Fructose and mannose metabolism \| \| \| --- \| --- \| --- \| --- \| --- \| --- \| --- \| --- \| --- \| | SMU_1185, SMU_1426c, SMU_100, SMU_1077, SMU_1877, SMU_1960,  SMU_2038, SMU_2047, SMU_435,  SMU_980, SMU_99 |
|  | **II**  **(25 genes)** | **1.73** | \| GOTERM_BP_FAT \| GO:0006350~transcription \| \| --- \| --- \| \| GOTERM_BP_FAT \| GO:0045449~regulation of transcription \| \| GOTERM_MF_FAT \| GO:0003700~transcription factor activity \| \| GOTERM_BP_FAT \| GO:0051252~regulation of RNA metabolic process \| \| GOTERM_BP_FAT \| GO:0006355~regulation of transcription, DNA-dependent \| \| SP_PIR_KEYWORDS \| transcription regulation \| \| GOTERM_MF_FAT \| GO:0030528~transcription regulator activity \| \| INTERPRO \| IPR012287:Homeodomain-related \| \| SP_PIR_KEYWORDS \| Transcription \| \| GOTERM_MF_FAT \| GO:0003677~DNA binding \| \| SP_PIR_KEYWORDS \| DNA-binding \| \| INTERPRO \| IPR011991:Winged helix repressor DNA-binding \| | *hrcA, ptsH, ccpA, rex, xerS*, SMU_1027, SMU_1029, SMU_1165c, SMU_1287, SMU_134, SMU_135, SMU_136c, SMU_1397c, SMU_1409c, SMU_1599, SMU_1604c, SMU_173, SMU_1977c, SMU_1995c, SMU_207c, SMU_226c, SMU_236c, SMU_441, SMU_491, SMU_661 |
|  | **III**  **(6 genes)** | **1.64** | \| SMART \| SM00530:HTH_XRE \| \| --- \| --- \| \| INTERPRO \| IPR001387:Helix-turn-helix type 3 \| \| GOTERM_MF_FAT \| GO:0043565~sequence-specific DNA binding \| | SMU_136c, SMU_1397c,  SMU_1409c,  SMU_1977c,  SMU_207c,  SMU_661 |
|  | **IV**  **(27 genes)** | **1.49** | \| GOTERM_MF_FAT \| GO:0008982~protein-N(PI)-phosphohistidine-sugar PTS activity \| \| --- \| --- \| \| GOTERM_BP_FAT \| GO:0009401~phosphoenolpyruvate-dependent sugar PTS \| \| GOTERM_BP_FAT \| GO:0008643~carbohydrate transport \| \| KEGG_PATHWAY \| smu02060:Phosphotransferase system (PTS) \| \| SP_PIR_KEYWORDS \| Phosphotransferase system \| \| INTERPRO \| IPR003352:Phosphotransferase system, EIIC \| \| GOTERM_MF_FAT \| GO:0005351~sugar:hydrogen symporter activity \| \| GOTERM_MF_FAT \| GO:0005402~cation:sugar symporter activity \| \| GOTERM_MF_FAT \| GO:0015295~solute:hydrogen symporter activity \| \| GOTERM_MF_FAT \| GO:0051119~sugar transmembrane transporter activity \| \| INTERPRO \| IPR018113:PTS EIIB/cysteine phosphorylation site \| \| INTERPRO \| IPR001127:PTS, sugar-specific permease EIIA 1 domain \| \| INTERPRO \| IPR001996:Phosphotransferase system, EIIB \| \| INTERPRO \| IPR013013:Phosphotransferase system, EIIC component, type 1 \| \| GOTERM_MF_FAT \| GO:0015293~symporter activity \| \| GOTERM_MF_FAT \| GO:0015294~solute:cation symporter activity \| \| SP_PIR_KEYWORDS \| sugar transport \| \| GOTERM_CC_FAT \| GO:0031224~intrinsic to membrane \| \| GOTERM_CC_FAT \| GO:0016021~integral to membrane \| \| SP_PIR_KEYWORDS \| kinase \| \| SP_PIR_KEYWORDS \| transferase \| \| GOTERM_CC_FAT \| GO:0005886~plasma membrane \| \| SP_PIR_KEYWORDS \| transport \| \| SP_PIR_KEYWORDS \| cell membrane \| \| SP_PIR_KEYWORDS \| transmembrane \| \| SP_PIR_KEYWORDS \| membrane \| | *glgB,*  *rmlA,*  *mtlA1,*  *ptsH,*  SMU_2162c,  *prmA*,  SMU_100,  SMU_1028,  SMU_1030,  SMU_1299c,  SMU_1599,  SMU_180,  SMU_1848,  SMU_1877,  SMU_1948,  SMU_1957,  SMU_1958c,  SMU_1960c,  SMU_2038,  SMU_2047,  SMU_2151,  SMU_24 ,  *lrgA,*  SMU_980,  *lacC,*  *upp,*  *wapA* |
| **UP-regulated**  **(Normal/**  **Simulated micro-G)** | **V**  **(12 genes)** | **1.32** | \| GOTERM_BP_FAT \| GO:0006525~arginine metabolic process \| \| --- \| --- \| \| KEGG_PATHWAY \| smu00330:Arginine and proline metabolism \| \| SP_PIR_KEYWORDS \| arginine biosynthesis \| \| GOTERM_BP_FAT \| GO:0006526~arginine biosynthetic process \| \| GOTERM_BP_FAT \| GO:0009064~glutamine family amino acid metabolic process \| \| GOTERM_BP_FAT \| GO:0009084~glutamine family amino acid biosynthetic process \| \| GOTERM_BP_FAT \| GO:0044271~nitrogen compound biosynthetic process \| \| GOTERM_BP_FAT \| GO:0009309~amine biosynthetic process \| \| SP_PIR_KEYWORDS \| amino-acid biosynthesis \| \| GOTERM_BP_FAT \| GO:0008652~cellular amino acid biosynthetic process \| \| GOTERM_BP_FAT \| GO:0046394~carboxylic acid biosynthetic process \| \| GOTERM_BP_FAT \| GO:0016053~organic acid biosynthetic process \| | *argB,*  *argD,*  *argJ,*  *pyrA,*  *pyrK,*  SMU_915c,  SMU_264,  *tgt,*  *queC,*  *otcA,*  *arc,*  SMU_917c |
|  | **VI**  **(7 genes)** | **1.30** | \| SP_PIR_KEYWORDS \| queuosine biosynthesis \| \| --- \| --- \| \| GOTERM_BP_FAT \| GO:0046118~7-methylguanosine biosynthetic process \| \| GOTERM_BP_FAT \| GO:0008616~queuosine biosynthetic process \| \| GOTERM_BP_FAT \| GO:0008618~7-methylguanosine metabolic process \| \| GOTERM_BP_FAT \| GO:0046116~queuosine metabolic process \| \| GOTERM_BP_FAT \| GO:0046114~guanosine biosynthetic process \| \| GOTERM_BP_FAT \| GO:0046128~purine ribonucleoside metabolic process \| \| GOTERM_BP_FAT \| GO:0042278~purine nucleoside metabolic process \| \| GOTERM_BP_FAT \| GO:0046129~purine ribonucleoside biosynthetic process \| \| GOTERM_BP_FAT \| GO:0009163~nucleoside biosynthetic process \| \| GOTERM_BP_FAT \| GO:0042451~purine nucleoside biosynthetic process \| \| GOTERM_BP_FAT \| GO:0042455~ribonucleoside biosynthetic process \| \| GOTERM_BP_FAT \| GO:0009119~ribonucleoside metabolic process \| \| GOTERM_BP_FAT \| GO:0008617~guanosine metabolic process \| \| GOTERM_BP_FAT \| GO:0008033~tRNA processing \| \| GOTERM_BP_FAT \| GO:0006400~tRNA modification \| \| GOTERM_BP_FAT \| GO:0034470~ncRNA processing \| \| GOTERM_BP_FAT \| GO:0009116~nucleoside metabolic process \| \| GOTERM_BP_FAT \| GO:0009451~RNA modification \| \| GOTERM_BP_FAT \| GO:0034404~nucleobase, nucleoside and nucleotide biosynth. process \| \| GOTERM_BP_FAT \| GO:0034654~nucleobase, nucleoside, nucleotide and nucleic acid biosynth. \| \| GOTERM_BP_FAT \| GO:0006396~RNA processing \| \| GOTERM_BP_FAT \| GO:0006399~tRNA metabolic process \| \| GOTERM_BP_FAT \| GO:0034660~ncRNA metabolic process \| | *pyrA,*  *pyrK,*  *trmFO,*  SMU_915c,  *tgt,*  *queC,*  SMU_942 |

**Table S3. qPCR primers used in this study**

| **Primer** | **Sequence (5'-3')** |
| --- | --- |
| 1547-F | GGTTTACCAGGTGAAAGCCA |
| 1547-R | CGCGTATTGGTCATGAGATG |
| 2003a-F | ACCATCTGTTAAACCAATTTGCG |
| 2003a-R | TGGCGTTGTTTGTGTTTTGG |
| 669-F | TAGGATTTACTGCCGCTCCTG |
| 669-R | TCAATTTAGCAGGCTGAAACCC |
| 917c-F | TTGTCCCAAACGCGTTCTTG |
| 917c-R | CCGCGTTCATCAAGAAAAGC |
| 1296-F | ACTGGCTCTTCTGGCAAATG |
| 1296-R | ATTGGCGCTTGGTTTCCATG |
| mazF-F | GCGCCAATTAGCAATACCAAGC |
| mazF-R | TCCCTGTGGATTCTGTTCCTTC |
| wapA-F | CGACAACTAGTGAAGCGACAAC |
| wapA-R | TGTTGAAGCTCCTGTTGTGC |
| 1053-F | TGGCAATATTGGTCGTGCTC |
| 1053-R | ACCGTCACTTGTCGTTTGAC |
| 1291c-F | AGCAGGCCTTGTTGTCAATG |
| 1291c-R | CCCCATCTAATGCTCTCCTTTG |
| ptcB-F | ATGCGTTTTTGCCCTTCTGG |
| ptcB-R | GCTAAAAAGACGATGGCCCTTC |
| lrgA-F | TTGCCTAAAGCCTTACCGATTCC |
| lrgA-R | GCCTGATGGGACAAACATAAAGC |
| gyrB-F | ACGTTCAAAACCGACCGTTC |
| gyrB-R | ATGAAACGCGTGCCATCAAG |
