# Supplementary material for: Investigation of simulated microgravity effects on Streptococcus mutans physiology and global gene expression
Source: NPJ Microgravity. 2017 Jan 12;3:4. doi: 10.1038/s41526-016-0006-4 (PMC5460135; doi:10.1038/s41526-016-0006-4)
Supplement: Supplementary file 2 — Supplementary Figures [file 41526_2016_6_MOESM2_ESM.pdf]

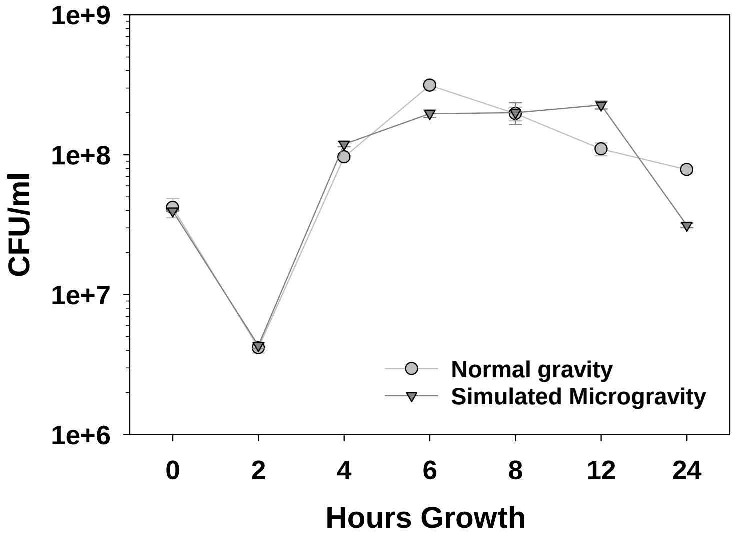

Figure S1. Continuous growth curve analysis of *S. mutans* simulated microgravity and normal gravity HARV cultures. Each simulated microgravity (triangles) and normal gravity (circles) HARV growth curve was performed in biofilm media for 24 hours at 37°C and 5% CO2. HARVs were sampled for serial dilution and CFU/ml determination at t = 0 (time of inoculation), 2, 4, 6, 8, 12, and 24 hours post-inoculation, as described in Materials and Methods. Data represent n=3 independent experiments per growth condition, error bars = standard error of the mean (SEM).

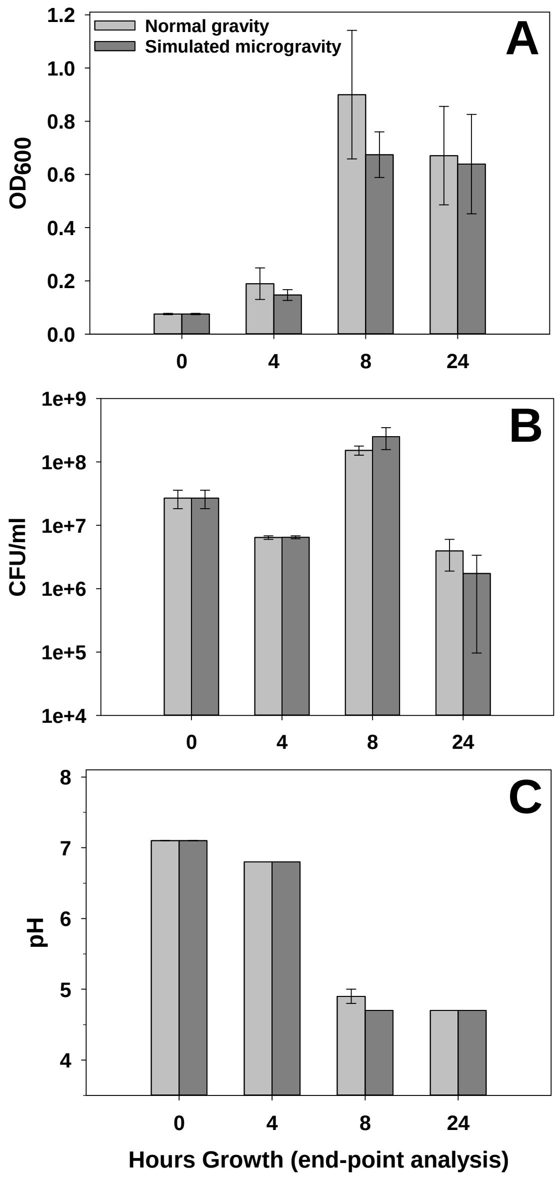

Figure S2. OD_{600} (A), CFU/ml (B), and pH (C) data corresponding to end-point *S. mutans* microgravity and normal gravity HARV cultures harvested for metabolomics (t=4,8, and 24 hours growth) and for RNA-seq (t=8 hours only). Data represent n=3 independent experiments per time point per growth condition. Error bars = standard error of the mean (SEM).

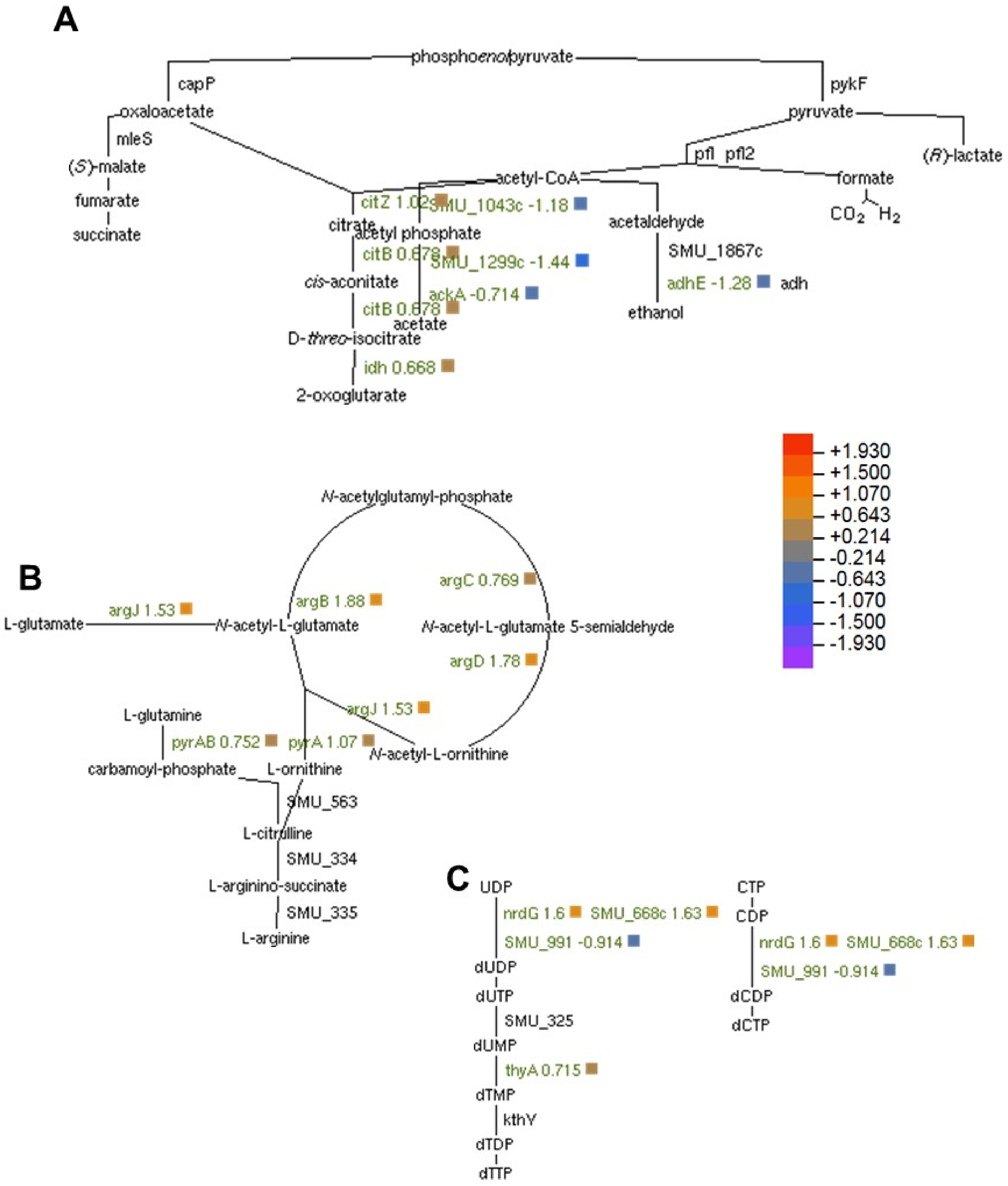

Figure S3. Metacyc analysis of RNA-seq data. The top three metabolic pathways to which statistically-significant differentially-expressed genes matched were mixed-acid fermentation (A), arginine biosynthesis (acetyl cycle) (B), and pyrmidine deoxynucleotide de novo biosynthesis (C). Heat-map indicates log2-fold change in gene expression (normal gravity/simulated microgravity).

A

B

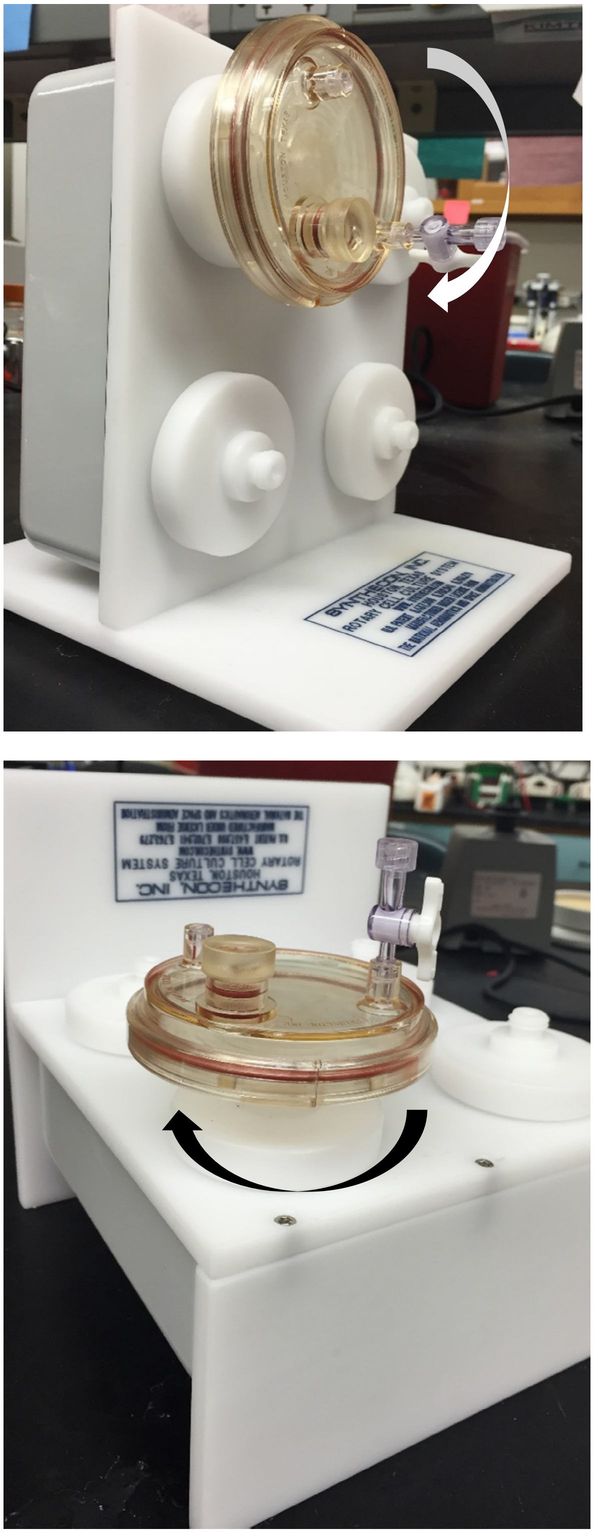

Figure S4. Diagrams of Disposable Vessel Rotary Cell Culture Systems used to simulate microgravity (A; HARV rotated on an axis perpendicular to gravitational vector) and normal gravity (B; HARV rotated on an axis parallel to gravitational vector) growth conditions.
