# Supplementary material for: Investigation of simulated microgravity effects on Streptococcus mutans physiology and global gene expression
Source: NPJ Microgravity. 2017 Jan 12;3:4. doi: 10.1038/s41526-016-0006-4 (PMC5460135; doi:10.1038/s41526-016-0006-4)
Supplement: Supplementary file 3 — Supplementary File S1 [file 41526_2016_6_MOESM3_ESM.docx]

**Supplemental Materials and Methods**

**Metabolite extraction and analysis:** Samples were prepared according to previously-published protocols ([1-3](#_ENREF_1)). Briefly, an automated liquid handler (Hamilton LabStar, Salt Lake City, UT) was used to add methanol containing recovery standards to the experimental samples to facilitate protein precipitation. Following centrifugation, the supernatants were split into four aliquots for analysis on the three platforms, with one aliquot retained as a spare. All aliquots were dried under nitrogen and vacuum-desiccated. The samples were subsequently reconstituted in 50 μL 0.1% formic acid in water (acidic conditions) or in 50 μL 6.5mM ammonium bicarbonate in water, pH 8 (basic conditions) for the UHPLC/MS/MS analyses or derivatized to a final volume of 50μL for GC/MS analysis using equal parts bistrimethyl-silyl-trifluoroacetamide and solvent mixture acetonitrile:dichloromethane:cyclohexane (5:4:1) with 5% triethylamine at 60°C for one hour. In addition, three types of controls were analyzed in concert with the experimental samples: aliquots of a “client matrix” (formed by pooling a small amount of each sample) served as technical replicates throughout the dataset, extracted water samples served as process blanks, and a cocktail of standards spiked into every analyzed sample allowed instrument performance monitoring. Experimental samples and controls were randomized across all platform run days.

The non-targeted metabolic profiling platform employed for this analysis combined three independent platforms: ultrahigh performance liquid chromatography/tandem mass spectrometry (UHPLC/MS/MS) optimized for basic species, UHPLC/MS/MS optimized for acidic species, and gas chromatography/mass spectrometry (GC/MS). For the UHLC/MS/MS analysis, aliquots were separated using a Waters Acquity UPLC (Waters, Millford, MA) and analyzed using a Q-Exactive high resolution/accurate mass spectrometer (Thermo Fisher Scientific, Inc., Waltham, MA) which consisted of an electrospray ionization (ESI) source and Orbitrap mass analyzer. Derivatized samples for GC/MS were separated on a 5% phenyldimethyl silicone column with helium as the carrier gas and a temperature ramp from 60°C to 340°C and then analyzed on a Thermo-Finnigan Trace DSQ MS (Thermo Fisher Scientific, Inc.) operated at unit mass resolving power with electron impact ionization and a 50-750 atomic mass unit scan range.

Metabolites were identified by automated comparison of the ion features in the experimental samples to a reference library of chemical standard entries that included retention time, molecular weight (*m/z*), preferred adducts, and in-source fragments as well as associated MS spectra, and were curated by visual inspection for quality control using software developed at Metabolon ([4](#_ENREF_4)).

For data display purposes and statistical analysis, each biochemical was rescaled to set the median equal to 1. In addition, any missing values were assumed to be below the limits of detection and these values were imputed with the compound minimum (minimum value imputation). Following median scaling and imputation of missing values, statistical analysis of log-transformed data was performed using “R” (http://cran.r-project.org/), which is a freely available, open-source software package. Biochemicals that differed significantly between the experimental groups were determined using a two-way ANOVA test. *P*-values ≤0.05 were considered statistically significant and *p*-values <0.10 were reported as trends. Multiple comparisons were accounted for by estimating the false discovery rate (FDR) using q-values ([5](#_ENREF_5)). Principal Components Analysis (PCA) was also applied to the metabolomics data.

**qPCR validation of RNAseq data:** A subset of genes that were identified as differentially-expressed in the RNAseq experiment were validated by quantitative real-time PCR (qPCR) as previously-described ([6](#_ENREF_6)), using the primers listed in Supplemental Table S3 and the Livak method ([7](#_ENREF_7)) to calculate fold-change expression (*gyrB* was used as the reference gene). The fold-change values for qPCR results are expressed as log_2_ fold-change in Supplemental Table S1.

References:

1. Evans AM, DeHaven CD, Barrett T, Mitchell M, Milgram E. Integrated, nontargeted ultrahigh performance liquid chromatography/electrospray ionization tandem mass spectrometry platform for the identification and relative quantification of the small-molecule complement of biological systems. Analytical chemistry. 2009 Aug 15;81(16):6656-67. PubMed PMID: 19624122.

2. Evans CR, Karnovsky A, Kovach MA, Standiford TJ, Burant CF, Stringer KA. Untargeted LC-MS metabolomics of bronchoalveolar lavage fluid differentiates acute respiratory distress syndrome from health. Journal of proteome research. 2014 Feb 7;13(2):640-9. PubMed PMID: 24289193. Pubmed Central PMCID: 4068805.

3. Ohta T, Masutomi N, Tsutsui N, Sakairi T, Mitchell M, Milburn MV, et al. Untargeted metabolomic profiling as an evaluative tool of fenofibrate-induced toxicology in Fischer 344 male rats. Toxicologic pathology. 2009 Jun;37(4):521-35. PubMed PMID: 19458390.

4. Dehaven CD, Evans AM, Dai H, Lawton KA. Organization of GC/MS and LC/MS metabolomics data into chemical libraries. Journal of cheminformatics. 2010;2(1):9. PubMed PMID: 20955607. Pubmed Central PMCID: 2984397.

5. Storey JD, Tibshirani R. Statistical significance for genomewide studies. Proceedings of the National Academy of Sciences of the United States of America. 2003 Aug 5;100(16):9440-5. PubMed PMID: 12883005. Pubmed Central PMCID: 170937.

6. Lewis AM, Rice KC. Quantitative Real-Time PCR (qPCR) Workflow for analyzing *Staphylococcus aureus* gene expression. Methods in Molecular Biology. 2015 Feb 3. PubMed PMID: 25646613.

7. Livak KJ, Schmittgen TD. Analysis of relative gene expression data using real-time quantitative PCR and the 2(-Delta Delta C(T)) Method. Methods. 2001 Dec;25(4):402-8. PubMed PMID: 11846609.
